# Oxygen utilization during moderate-intensity resistance and aerobic exercise in arrhythmogenic cardiomyopathy: the central role of the periphery

**DOI:** 10.1093/eschf/xvag163

**Published:** 2026-06-05

**Authors:** Simon Wernhart, Manuel Rattka, Marwin Shir, Karl-Ludwig Laugwitz, Martin Halle, Mark J Haykowsky

**Affiliations:** Department for Preventive Sports Medicine and Sports Cardiology, TUM School of Medicine and Health, TUM University Hospital, Technical University of Munich (TUM), Ismaningerstrasse 22, Munich 81675, Germany; DZHK (German Centre for Cardiovascular Research), partner site Munich Heart Alliance, Munich, Marchioninistraße 15, 81377 Munich, Germany; TUM School of Medicine and Health, Department of Internal Medicine I, Technical University of Munich, University Hospital, Munich, Germany; Department for Preventive Sports Medicine and Sports Cardiology, TUM School of Medicine and Health, TUM University Hospital, Technical University of Munich (TUM), Ismaningerstrasse 22, Munich 81675, Germany; DZHK (German Centre for Cardiovascular Research), partner site Munich Heart Alliance, Munich, Marchioninistraße 15, 81377 Munich, Germany; TUM School of Medicine and Health, Department of Internal Medicine I, Technical University of Munich, University Hospital, Munich, Germany; Department for Preventive Sports Medicine and Sports Cardiology, TUM School of Medicine and Health, TUM University Hospital, Technical University of Munich (TUM), Ismaningerstrasse 22, Munich 81675, Germany; DZHK (German Centre for Cardiovascular Research), partner site Munich Heart Alliance, Munich, Marchioninistraße 15, 81377 Munich, Germany; Integrated Cardiovascular and Exercise Physiology and Rehabilitation (iCARE) Laboratory, College of Health Sciences, University of Alberta, Edmonton, Alberta, Canada; Department of Cardiology, Medizin Campus Hochgebirgsklinik, Davos, Switzerland

**Keywords:** Arrhythmogenic cardiomyopathy, Exercise, Oxygen uptake, Oxygen transport

## Abstract

**Aims:**

To quantify central oxygen delivery (O_2_D), peripheral oxygen extraction, and muscle diffusive oxygen conductance (DmO_2_) during upper-extremity resistance and lower-extremity aerobic exercise in patients with arrhythmogenic cardiomyopathy (ACM).

**Methods and results:**

Nineteen patients with ACM underwent invasive cardiopulmonary exercise testing with right heart and radial artery catheterization. Participants performed 1-min isometric handgrip (IM-HG) and bicep curl (BC) exercise at 70% maximal voluntary contraction, and 20- and 40-min supine cycling (CYC-20, CYC-40) at the first ventilatory threshold. Exercise pulmonary hypertension, defined by a cardiac output to mean pulmonary artery slope >3 mmHg/L/min, did not occur (mean 0.67 mmHg/L/min). Whole-body oxygen uptake (V̇O_2_) increased significantly across all modes (*P* < .001); however, central O_2_D did not increase significantly during IM-HG or BC and rose only modestly during cycling. In contrast, peripheral O_2_ extraction and DmO_2_ increased significantly across all modalities (*P* < .001). Dominance analysis revealed that DmO_2_ accounted for 77% of the variance in V̇O_2_ pooled across all conditions, whereas O_2_D accounted for only 23%.

**Conclusions:**

Moderate-intensity isometric and dynamic resistance and aerobic exercise in ACM is mediated by a predominantly peripheral, rather than central, physiological stress in our small ACM sample. This may provide preliminary results for a physiological rationale for safe exercise in this population.

## Background

Exercise recommendations for individuals with arrhythmogenic cardiomyopathy (ACM) are limited to 150 min of mild–moderate intensity exercise per week due to concerns that exercise-induced haemodynamic stress may provoke ventricular arrhythmia and accelerate disease progression.^1^ This recommendation is partially based on the assumption that the increased metabolic demands of exercise are meditated by a proportional increase in central oxygen delivery (O_2_D), thereby imposing significant mechanical stress on the fragile myocardium.^2,3^ However, whole-body oxygen uptake (V̇O_2_) is determined by both central O_2_D and peripheral factors, specifically fractional oxygen extraction and muscle diffusive oxygen conductance (DmO_2_).^4,5^ Currently, the relative contributions of these determinants during small muscle mass isometric and resistance exercise and large muscle mass aerobic exercise in ACM remain unknown.

## Aims

This study aimed to quantify central O_2_D, peripheral oxygen extraction, and DmO_2_ during upper-extremity resistance and isometric exercise and lower-extremity aerobic exercise in patients with ACM. We hypothesized that increases in V̇O_2_ across all modes of exercise would be driven predominantly by peripheral factors rather than central O_2_D across all exercise modalities.

## Methods

Nineteen patients with a prior diagnosis of ACM (43.8 ± 9.3 years; seven male) underwent invasive cardiopulmonary exercise testing. As previously described,^6^ on Day 1, participants completed maximal voluntary contraction (MVC) testing for isometric handgrip (IM-HG) and bicep curl (BC) one-repetition maximum (1RM) testing, followed by a symptom-limited supine cardiopulmonary exercise test to determine the first ventilatory threshold (VnT1). Resting echocardiography was performed to assess left and right ventricular ejection fraction. On Day 2, participants performed 1-min IM-HG and BC exercises at 70% MVC and 1RM, as well as 20- and 40-min continuous supine cycling (CYC-20, CYC-40) at a workload corresponding to VnT1 in a non-randomized sequence. A 5-min rest period separated each modality.

During Day 2, invasive right heart and radial artery catheterization were utilized to continuously measure haemodynamics and obtain blood samples at rest and at the end of each exercise modality.^6^ V̇O_2_ was calculated using the direct Fick principle. Mean capillary oxygen partial pressure (Pcap) was determined by forward (Bohr) integration as previously described.^7^ DmO_2_ was calculated as V̇O_2_ divided by the gradient between Pcap and a constant mitochondrial oxygen partial pressure of 3.1 mmHg.^8^ Fractional oxygen extraction was calculated as the arterial-mixed venous oxygen content difference divided by arterial oxygen content. O_2_D was calculated as the product of cardiac output and arterial oxygen content.^7^

Differences across the five conditions (Rest, IM-HG, BC, CYC-20, CYC-40) were assessed using repeated-measures ANOVA or the Friedman test, with Bonferroni-corrected post-hoc comparisons. Dominance analysis based on the all-subsets *R*^2^ approach was performed to quantify the relative contributions of central (O_2_D) and peripheral (DmO_2_) factors to the variance in V̇O_2_. Statistical significance was set at *P* < .05.

Beta-blockers and all regular medication were given in the morning of the exam. Antiarrhythmic drugs had been titrated previously to the maximal tolerable dose. During the study, no dose adjustments were made. No patient had received concomitant flecainide, sotalol, or amiodarone.

## Results

Left (58.8 ± 9.4%) and right (40.3 ± 11.3%) ventricular ejection fraction was predominantly preserved. Fourteen patients harboured plakophilin-2 (PKP2) variants, one patient had a pathogenic Filamin C variant, two patients carried variants of unknown significance in the desmin and desmoplakin genes (each *n* = 1). Two patients displayed a negative genotype. Most patients displayed right (*n* = 11), rather than left (*n* = 1) or biventricular (*n* = 7) involvement. Twelve patients had implantable cardioverter-defibrillators (ICD) and 14 received beta-blockers. Four patients with ICD received successful right ventricular ablation therapy for recurrent ventricular tachycardia prior to the study with no recurrence of arrhythmia under the established drug therapy. Ten patients displayed late gadolinium enhancement on magnetic resonance imaging (two ring-like patterns, one midventricular septal, seven right ventricular involvement of the free wall and the apex). Six patients had received secondary prophylactic ICDs (*n* = 2 for aborted sudden cardiac death, *n* = 4 for sustained ventricular tachycardia), while six received primary prophylactic implantation (high risk due to clinical decision and/or gene-specific risk calculation).

**Figure 1.**
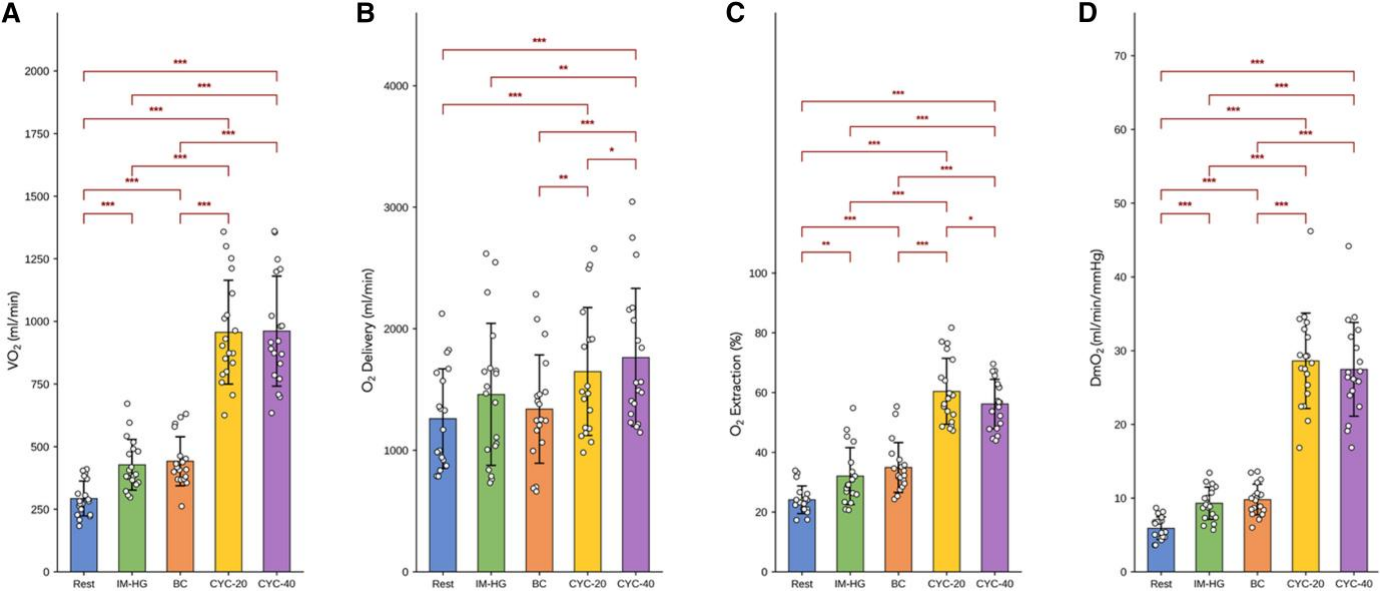
Oxygen transport and utilization at rest and during isometric handgrip (IM-HG), bicep curl (BC) and cycling at 20 (CYC-20) and 40 (CYC-40) minutes in patients with arrhythmogenic cardiomyopathy. Panels depict: (A) Oxygen utilization (V̇O_2_), (B) Oxygen delivery (O_2_D), (C) O_2_ extraction, and (D) Muscle diffusive oxygen conductance (DmO_2_). Significance brackets indicate pairwise comparisons (Bonferroni-corrected): *P < .05, **P < .01, ***P < .001

As shown in *Figure 1A*, all exercise modes significantly increased in V̇O_2_ (Friedman χ^2^(4) = 68.67, *P* < .001). Arm exercise produced modest rises of ∼52% (both IM-HG and BC *P* < .001 vs rest), while cycling was associated with a marked (∼233%) increase in V̇O_2_ (both CYC-20 and CYC-40 *P* < .001 vs rest). V̇O_2_ was not significantly different between arm modes or cycling durations. Exercise pulmonary hypertension, defined by a cardiac output to mean pulmonary artery slope >3 mmHg/L/min, did not occur (mean 0.65 mmHg/L/min). Cardiac output increased from rest (7.1 ± 1.9 L/min) to IM-HG (7.9 ± 2.7 L/min), BC (7.2 ± 2.1 L/min), CYC-20 (8.8 ± 2.4 L/min), and CYC-40 (9.5 ± 2.5 L/min; *P* < .001).

Despite a significant overall effect (*P* < .001), central O_2_D demonstrated a markedly blunted response. O_2_D did not increase significantly from rest during IM-HG (*P* = .272) or BC (*P* = 1.000, *Figure 1B*). Significant increases were confined to cycling: +32.3% at CYC-20 and +42.1% at CYC-40 (both *P* < .001 vs rest, *Figure 1B*). In contrast, peripheral O_2_ extraction and DmO_2_ increased significantly during all exercise modes (*Figure 1C–D*). Specifically, O_2_ extraction increased from 24% at rest to 32% (IM-HG) and 35% (BC, both *P* < .01), and more than doubled during cycling (CYC-20: 60%; CYC-40: 56%; both *P* < .001). DmO_2_ increased from rest by ∼69% during arm exercise (both *P* < .001) and ∼four-fold during cycling (both *P* < .001).

Dominance analysis using DmO_2_ and O_2_D as predictors explained 98.5% of the variance in V̇O_2_ pooled across all conditions. DmO_2_ was the dominant predictor, accounting for 77% of the explained variance, whereas O_2_D accounted for only 23%. This peripheral dominance was consistent within each exercise mode (DmO_2_ dominance: 50%–59%).

Continuous ECG monitoring was performed during right heart catheterization and revealed no clinically relevant arrhythmia. ICD interrogation was done in all patients at hospital discharge and confirmed that no ventricular arrhythmia occurred after exercise right heart catheterization. Non-ICD carriers received Holter monitoring after right heart catheterization, with one patient showing asymptomatic non-sustained ventricular tachycardia of six beats 20 h after invasive testing. This patient had already shown sustained ventricular tachycardia previously, independent of exercise but had declined ICD implantation.

## Conclusion

This study provides the first invasive quantification of the central and peripheral determinants of V̇O_2_ uptake during moderate-intensity upper and lower extremity exercise in patients with ACM. Our preliminary findings suggest that moderate-intensity resistance or aerobic exercise imposes a considerable peripheral contribution to exercise capacity in a heterogenous cohort of ACM patients.^1^ Across all modalities, the increased metabolic demand was met by marked increases in peripheral O_2_ extraction and DmO_2_. Moreover, dominance analysis confirmed that peripheral diffusion accounted for over three-quarters of the variance in V̇O_2_, relegating central delivery to a secondary role. During upper-extremity small muscle mass (IM-HG, BC) exercise, the central haemodynamic burden was minimal, with the stress localized almost entirely to the exercising skeletal muscle. Moreover, during CYC-20 and 40, O_2_D increased 32 to 42% while DmO_2_ increased four-fold. Collectively, our preliminary data suggest that moderate-intensity aerobic and resistance exercise appears safe as no significant adverse events were observed. However, causality of one six-beat non-sustained ventricular tachycardia 20 h after testing cannot be established as this patient had previously shown sustained ventricular tachycardia, independent of exercise. By maintaining peripheral fitness and extraction efficiency, such programmes may chronically reduce the central cardiac stress required during submaximal exercise, thereby improving functional capacity without accelerating disease progression. However, it is important to note that these findings reflect only acute exercise responses, and the long-term effects of such interventions require more comprehensive evaluation.

Our cohort showed predominantly preserved left and right ventricular ejection fraction and primarily consisted of PKP2-variants with a right-dominant phenotype, which is a major limitation but is comparable to other exercise studies in ACM.^9^ The results cannot be generalized to patients with advanced ACM, left-dominant phenotypes, severe ventricular dysfunction, or higher arrhythmic burden. Most patients received centrally acting beta-blockers, which may not have an impact on diffusion capacity. However, five patients did not receive any beta-blockers, which limits generalizability of our results. CYC-20 can be regarded as the ‘daily dose’ of the 150 min of weekly exercise recommended in ACM.^1^ Importantly, extending exercise duration, but not intensity, to the double dose (CYC-40), did not change the central haemodynamic load. Thus, modestly extending the currently recommended duration of moderate-intensity aerobic exercise may be feasible, although this requires confirmation through long-term follow-up in this well-characterized ACM cohort. In addition, both small-muscle-mass isometric and dynamic moderate-intensity resistance exercises are associated with predominantly peripheral rather than central physiological stress. This observation warrants further investigation in a prospective, adequately powered multicentre trial to determine whether such training can preserve cardiorespiratory fitness without increasing the risk of premature heart failure or malignant arrhythmias. Taken together, our findings provide additional physiological support for the recently proposed view that small-muscle-mass resistance training may be a reasonable option for patients with ACM.^10^

We acknowledge several limitations of this study. First, the modest sample size, while substantial for an invasive catheterization study in a rare disease population, limits generalizability across the full spectrum of ACM phenotypes, genotypes, and disease severities. Our results cannot be generalized to patients with left-dominant, non-PKP-2 variants, severely reduced ejection fraction, or significant arrhythmic burden. Also, we cannot provide a group of healthy individuals as a direct comparator. Performing exercise right heart catheterization in healthy individuals is not feasible in our institution due to ethical reasons. Although centrally acting beta-blockers were the predominantly prescribed antiarrhythmic drug, treatment heterogeneity persists and must be acknowledged. Second, all exercise protocols were performed in the supine position to facilitate invasive haemodynamic monitoring. This, however, augments venous return relative to upright exercise and may have modestly attenuated the central O_2_D response. Translation to upright exercise testing and derivation of exercise recommendations should be performed with caution. Third, modalities were sequential and non-randomized, which may have resulted in ‘carry-over’ effects for fatigue, cumulative sympathetic activation, and possible haemodynamic interactions between exercise stages. Future adequately powered studies should apply a randomized sequence of exercise modes to compensate for this limitation. Fourth, the mathematical dependency of D_m_O_2_ on V̇O_2_ must be acknowledged when interpreting the dominance analysis results. Finally, this study characterizes the acute physiological response to a single exercise bout; longitudinal training studies are needed to determine the effects of central and peripheral determinants of V̇O_2_ in ACM and establish whether our suggested exercise prescription approach translates into a reduction of adverse events.

Moderate-intensity IM-HG and BC exercise and cycle exercise displayed considerable peripheral physiological stress in our small and heterogenous ACM cohort. Across all modalities, the increased O_2_ was driven by increased peripheral oxygen extraction and muscle diffusive oxygen conductance. These findings provide a strong potential physiological rationale for moderate-intensity small muscle mass upper extremity and large muscle mass lower extremity aerobic exercise in ACM. Adequately powered studies are warranted to demonstrate whether this approach is safe across the spectrum of genotype–phenotype correlation of ACM patients.

## Declarations

### Data Availability

Data can be obtained from the corresponding author upon reasonable request.

## References

[1] Pelliccia A, Sharma S, Gati S, Bäck M, Börjesson M, Caselli S, et al 2020 ESC guidelines on sports cardiology and exercise in patients with cardiovascular disease. Eur Heart J 2021;42:17–96. 10.1093/eurheartj/ehaa605 32860412

[2] La Gerche A . Exercise-induced arrhythmogenic (right ventricular) cardiomyopathy is real…if you consider it. JACC Cardiovasc Imaging 2021;14:159–61. 10.1016/j.jcmg.2020.09.014 33221208

[3] La Gerche A, Claessen G, Dymarkowski S, Voigt JU, De Buck F, Vanhees L, et al Exercise-induced right ventricular dysfunction is associated with ventricular arrhythmias in endurance athletes. Eur Heart J 2015;36:1998–2010. 10.1093/eurheartj/ehv202 26038590

[4] Wagner PD . Determinants of maximal oxygen transport and utilization. Annu Rev Physiol 1996;58:21–50. 10.1146/annurev.ph.58.030196.000321 8815793

[5] Wagner PD . Diffusive resistance to O2 transport in muscle. Acta Physiol Scand 2000;168:609–14. 10.1046/j.1365-201x.2000.00712.x 10759597

[6] Wernhart S, Shir M, Federle D, Laugwitz KL, Halle M, Rattka M, et al Multi-site lactate kinetics during moderate intensity resistance and endurance exercise in definitive and probable arrhythmogenic cardiomyopathy. Int J Cardiol Heart Vasc 2026;64:101913. 10.1016/j.ijcha.2026.101913 41953325 PMC13054101

[7] Roca J, Agusti AG, Alonso A, Poole DC, Viegas C, Barbera JA, et al Effects of training on muscle O2 transport at VO2max. J Appl Physiol (1985) 1992;73:1067–76. 10.1152/jappl.1992.73.3.1067 1400019

[8] Broxterman RM, Wagner PD, Richardson RS. Endurance exercise training changes the limitation on muscle V̇O2max in normoxia from the capacity to utilize O(2) to the capacity to transport O(2). J Physiol 2024;602:445–59. 10.1113/JP285650 38048175 PMC10841684

[9] Ruwald AC, Marcus F, Estes NA 3rd, Link M, McNitt S, Polonsky B, et al Association of competitive and recreational sport participation with cardiac events in patients with arrhythmogenic right ventricular cardiomyopathy: results from the North American multidisciplinary study of arrhythmogenic right ventricular cardiomyopathy. Eur Heart J 2015;36:1735–43. 10.1093/eurheartj/ehv110 25896080 PMC4500847

[10] Dei LL, Han J, Romano S, Sciarra L, Asimaki A, Papadakis M, et al Exercise prescription in arrhythmogenic cardiomyopathy: finding the right balance between risks and benefits. JAHA 2025;14:e039125. 10.1161/JAHA.124.039125 40470644 PMC12229179

